# Potential Prognostic Biomarkers of NIMA (Never in Mitosis, Gene A)-Related Kinase (NEK) Family Members in Breast Cancer

**DOI:** 10.3390/jpm11111089

**Published:** 2021-10-26

**Authors:** Gangga Anuraga, Wei-Jan Wang, Nam Nhut Phan, Nu Thuy An Ton, Hoang Dang Khoa Ta, Fidelia Berenice Prayugo, Do Thi Minh Xuan, Su-Chi Ku, Yung-Fu Wu, Vivin Andriani, Muhammad Athoillah, Kuen-Haur Lee, Chih-Yang Wang

**Affiliations:** 1 PhD Program for Cancer Molecular Biology and Drug Discovery, College of Medical Science and Technology, Taipei Medical University and Academia Sinica, Taipei 11031, Taiwan; g.anuraga@unipasby.ac.id (G.A.); d621109004@tmu.edu.tw (H.D.K.T.); khlee@tmu.edu.tw (K.-H.L.); 2 Graduate Institute of Cancer Biology and Drug Discovery, College of Medical Science and Technology, Taipei Medical University, Taipei 11031, Taiwan; m142109005@tmu.edu.tw (F.B.P.); m654110001@tmu.edu.tw (D.T.M.X.); b101104152@tmu.edu.tw (S.-C.K.); 3 Department of Statistics, Faculty of Science and Technology, Universitas PGRI Adi Buana, Surabaya 60234, Indonesia; athoillah@unipasby.ac.id; 4 Research Center for Cancer Biology, Department of Biological Science and Technology, China Medical University, Taichung 40604, Taiwan; cvcsky@cmu.edu.tw; 5 Institute for Environmental Science, Nguyen Tat Thanh University, Ho Chi Minh City 700000, Vietnam; pnnam@ntt.edu.vn (N.N.P.); tntan@ntt.edu.vn (N.T.A.T.); 6 Department of Medical Research, Tri-Service General Hospital, School of Medicine, National Defense Medical Center, Taipei 11490, Taiwan; qrince@yahoo.com.tw; 7 Department of Biological Science, Faculty of Science and Technology, Universitas PGRI Adi Buana, Surabaya 60234, Indonesia; v.andriani@unipasby.ac.id; 8 Cancer Center, Wan Fang Hospital, Taipei Medical University, Taipei 11031, Taiwan

**Keywords:** breast cancer, bioinformatics, biomarker, prognosis, *NEK* family genes, immune microenvironment, immune infiltration, functional enrichment analysis

## Abstract

Breast cancer remains the most common malignant cancer in women, with a staggering incidence of two million cases annually worldwide; therefore, it is crucial to explore novel biomarkers to assess the diagnosis and prognosis of breast cancer patients. NIMA-related kinase (NEK) protein kinase contains 11 family members named NEK1-NEK11, which were discovered from *Aspergillus Nidulans*; however, the role of NEK family genes for tumor development remains unclear and requires additional study. In the present study, we investigate the prognosis relationships of NEK family genes for breast cancer development, as well as the gene expression signature via the bioinformatics approach. The results of several integrative analyses revealed that most of the NEK family genes are overexpressed in breast cancer. Among these family genes, *NEK2/6/8* overexpression had poor prognostic significance in distant metastasis-free survival (DMFS) in breast cancer patients. Meanwhile, *NEK2/6* had the highest level of DNA methylation, and the functional enrichment analysis from MetaCore and Gene Set Enrichment Analysis (GSEA) suggested that *NEK2* was associated with the cell cycle, G2M checkpoint, DNA repair, E2F, MYC, MTORC1, and interferon-related signaling. Moreover, Tumor Immune Estimation Resource (TIMER) results showed that the transcriptional levels of NEK2 were positively correlated with immune infiltration of B cells and CD4^+^ T Cell. Collectively, the current study indicated that *NEK* family genes, especially *NEK2* which is involved in immune infiltration, and may serve as prognosis biomarkers for breast cancer progression.

## 1. Introduction

Breast cancer is one of the most common cancers that frequently occurs in women. Moreover, it has become one of the significant causes of death in women throughout the world. According to the most recent global cancer burden report, 2.26 million new breast cancer cases were diagnosed globally [1]. Furthermore, the complexity of breast cancer makes it difficult to fully comprehend its carcinogenesis, progression, invasion, and metastasis using clinical and molecular markers used for early detection [2,3,4,5,6]. Therefore, it is crucial to explore potential novel biomarkers for assessing the diagnosis and prognosis of breast cancer patients [7,8,9,10,11,12].

NIMA (Never in Mitosis, Gene a)-Related Kinase (NEK) is a family of protein kinases [13]. NEK consists of 11 members of protein kinase, namely NEK1~NEK11 [14,15]. Detailed basic characteristics of the NEK gene family are presented in Table 1.

The protein kinase family of NEK has been implicated in the development of various cancers [13]. Previous research demonstrated that NEK1 regulated bladder [16], kidney [17], and breast cancer progression [18]. Several NEK genes have also been identified to be linked with breast cancer, such as NEK2 [19] and NEK3 [20] were found to be overexpressed in human breast cancer. MicroRNA (miR)-128-3p inhibits the stem-like cell characteristics of breast cancer stem cells (BCSCs) by inhibiting the Wnt signaling pathway via downregulating NEK2, creating a new target for breast cancer treatment [21]. NEK5 was also linked to breast cancer development and a poor prognosis [22]. NEK7 had high expression in larynx, breast, colorectal [23], and gall bladder cancers [24]. NEK8 is overexpressed in primary breast tumors in humans, and it has considerable sequence similarity to the NEK family of protein kinases and may be involved in G_2_/M development [25].

Various studies looking for novel polymorphisms in carriers of the BRCA type 1/2 susceptibility protein (BRCA1/2) mutation revealed that NEK10 mutations were associated with breast cancer [26,27]. Moreover, NEK10 phosphorylates p53 at Y327, promoting cell cycle arrest after exposure to DNA damaging agents [28]. NEK11 also plays an essential role in cancer development, and is required for survival, regardless of whether cells were exposed to DNA damage [29]. NEKs are associated with several DNA damage response pathways, such as ATM/ATR, CHK1, CDKs, p53/p21, and RAD51 [30]. NEK1 is an ATR activator that causes cell cycle arrest, ensuring DNA repair while also activating particular repair pathways, including homologous repair (HR) and DNA double-strand break (DSB) repair. ATR and ATM were downstream effectors of NEK2, 6, 8, 9, and 11, which results in cell cycle arrest, although the details of possibly active repair pathways are still being explored. Through recruitment of DNA-PK to DNA damage foci, NEK4 is linked to the control of non-homologous end-joining (NHEJ) repair of DNA DSBs [31,32,33,34,35,36].

**Figure 1:**
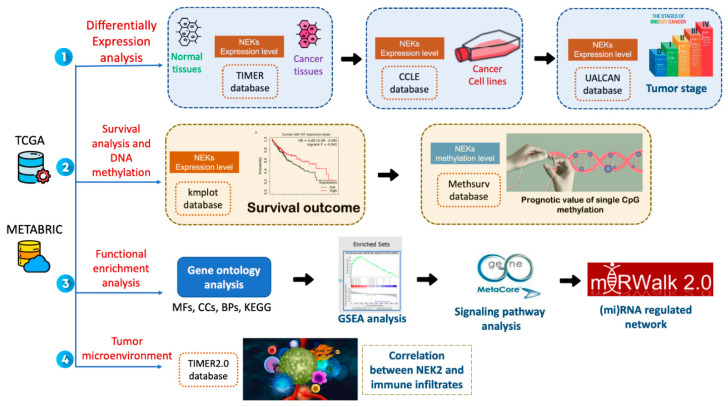
The workflow and study design of the analysis steps. TCGA: The Cancer Genome Atlas; METABRIC: Molecular Taxonomy of Breast Cancer International Consortium; NEK: NIMA-related kinase; GSEA: gene set enrichment analysis; BP: biological process; MF: Molecular Function; CC: Cellular Component; KEGG: Kyoto Encyclopedia of Genes and Genomes; CCLE: Cancer Cell Line Encyclopedia; MetaCore Analysis; micro (mi)RNA-regulated networks; TIMER: Tumor IMmune Estimation Resource.

Comprehensive analyses of the promising effects of the *NEK* gene family on breast cancer are lacking in the current stage. Furthermore, analyses with bioinformatics methods have not been widely used to investigate the performance of *NEK* family members in breast cancer. Cell cycle kinases play an essential role in the development of cancer therapy [37]. However, whether these *NEK* family members can regulate cell cycle kinase procedure is still unclear [38]. Therefore, a complete investigation of various members of the NEK family in breast cancer is needed to explain expression levels, molecular mechanisms, and functional enrichment analyses of breast cancer, which could potentially provide novel prognostic indicators for this complex disease (Figure 1). In this study, we used various large-scale bioinformatics databases to carry out systematic bioinformatics analysis [39,40,41,42,43]. First, we used the tumor immune estimation resource (TIMER) database, Cancer Cell Lines Encyclopedia (CCLE), and UALCAN analysis to determine *NEK* gene family expression differences between breast cancer and normal tissue. Second, we used Kaplan-Meier plots to reveal the significance of *NEK* gene family members to the prognosis of breast cancer patients. After that, we used MethSurv to determine the expression and prognostic patterns of single CpG methylation of the NEK gene family in breast cancer. Then, we studied the gene potential of the *NEK* family in depth through functional enrichment analysis and miRNA-regulated networks. This analysis was used to reveal the biological processes, molecular pathways, potentially regulated miRNAs, and their involvement in cell cycle kinases. In addition, we also used MetaCore to delve deeper into the enrichment pathways of potential *NEK* family genes in breast cancer development. Finally, we used the TIMER database to uncover the correlation of potential genes from the *NEK* family with immune cell markers in breast cancer. 

## 2. Materials and Methods

### 2.1. Expression Analysis of the NEK Genes Family

We used the tumor immune estimation resource (TIMER), Cancer Cell Lines Encyclopedia (CCLE), and UALCAN to determine differences in *NEK* gene family expressions between breast cancer and normal tissues. Through the DiffExp module in the TIMER database (https://cistrome.Shinyapps.io/timer/, accessed on 12 May 2020), we revealed differential expression patterns between normal and adjacent tumor tissues for each gene of interest in all TCGA tumors [44,45]. Furthermore, CCLE database (https://portals.broadinstitute.org/ccle, accessed on 12 May 2020) contains 1457 cancer cell lines (*n* = 1457) with 136,488 unique data (*n* = 136,488); therefore we used the CCLE to explore the expression of NEK family members in these cancer cell lines. UALCAN (http://ualcan.path.uab.edu, accessed on 12 May 2020) contains omics cancer data (TCGA, MET500, and CPTAC) [46], which we then used to investigate the expressions of *NEK* gene family members in normal and breast cancer tissues, and their clinicopathological significance. Furthermore, we used an independent-sample *t*-test to confirm the significance of expressions of *NEK* gene family members by normal and breast cancer tissues, and in terms of clinicopathological parameters. We used *p* < 0.05 for the threshold of concluding that there was a significant difference [47,48,49].

### 2.2. Survival Analysis of the NEK Genes Family

The KM plotter (http://kmplot.com/analysis/, accessed on 12 May 2020) has a dataset of about 54,000 genes [50], and survival information on several cancer types with a known sample of 7830 breast cancers (*n* = 7830) [51]. These data were used to explore the significance of *NEK* gene family members on distant metastasis-free survival (DMFS) in breast cancer patients. The hazard ratio (HR) with 95% confidence interval (CI) and log-rank *p* values were used to determine the significance of the overexpression of *NEK* family genes in terms of DMFS in breast cancer patients [52,53,54].

### 2.3. DNA Methylation of the NEK Genes Family

DNA methylation plays a vital role in prognostic assessment and potential biomarker in cancer development [55]. We used MethSurv (https://biit.cs.ut.ee/methsurv/, accessed on 12 May 2020) to determine the expression and prognostic patterns of single CpG methylation of the *NEK* gene family in breast cancer [56]. In this analysis, DNA methylation values are represented using beta values (beta values ranging from 0 to 1). Every single methylation of CpG was calculated by the M/(M + U + 100) formulation. M and U are methylated and unmethylated intensity values.

### 2.4. Functional Enrichment and Micro (mi)RNA-Regulated Networks Analysis

We used the Bioconductor “clusterProfiler” and “GOplot 1.0.2” packages in R Studio software [57,58] to test the potential functional significance of genes with molecular functions (MFs), cellular components (CCs), and biological processes (BPs) on GO and the Kyoto Encyclopedia of Genes and Genomes (KEGG) [59]. An adjusted *p* < 0.05 was chosen as the threshold for statistical significance. Next, we used the “fgsea” packages in R Studio software to evaluate enriched pathways in transcriptional data by a GSEA [60,61]. This study used a hallmark database analysis to display gene enrichment pathways in transcriptional data [62,63,64]. Furthermore, MetaCore (https://portal.genego.com/, accessed on 12 May 2020) was used to perform functional analyses on various omics data, which calculates the *p*-value of enrichment in different gene pools of an uploaded dataset [65,66,67,68,69]. Expression profiles of TCGA dataset on expressions of *NEK* gene family members were collected and in-depth integrated, by applying Venny vers. 2.1 (https://bioinfogp.cnb.csic.es/tools/venny/index.html, accessed on 12 May 2020), and results were subsequently uploaded to MetaCore for analysis. Finally, we also investigated the gene potential of the *NEK* family with miRNAs. We used miRWalk (http://mirwalk.umm.uni-heidelberg.de/, accessed on 12 May 2020) to investigate the regulatory potential of miRNAs, analyzing regulated pathways and networks by Ingenuity Pathway Analysis (IPA) [70,71,72,73,74].

### 2.5. Correlation Analysis between Gene Expressions and Immune Infiltration

TIMER 2.0 (http://timer.cistrome.org/, accessed on 12 May 2020) was used to examine the relationships among immune cells and various types of cancer [44,45]. This platform applies an algorithmic method to evaluate the abundances of immune cells that infiltrate tumor genes’ expression profiles. We investigated associations of potential expressions of *NEK* gene family members with the quantity of immune infiltrates in breast cancer through this dataset. The main objective was to find potential biomarkers at the level of immune infiltration in breast cancer tissues. We studied potential associations of gene expressions of the *NEK* family with various levels of infiltration such as purity, B cells, cluster of differentiation-positive (CD8^+^) T cells, CD4^+^ T cells, macrophages, neutrophils, and dendritic cells (DCs) [75,76]. The TIMER 2.0 analysis displays scatterplots and heatmaps to illustrate correlations between gene expressions and levels of immune infiltration. Next, we used Spearman correlation measures, *p* values, and adjusted *p* values to make statistical decisions.

## 3. Results

### 3.1. Expression Analysis of NEK Family Members in Breast Cancer

**Figure 2:**
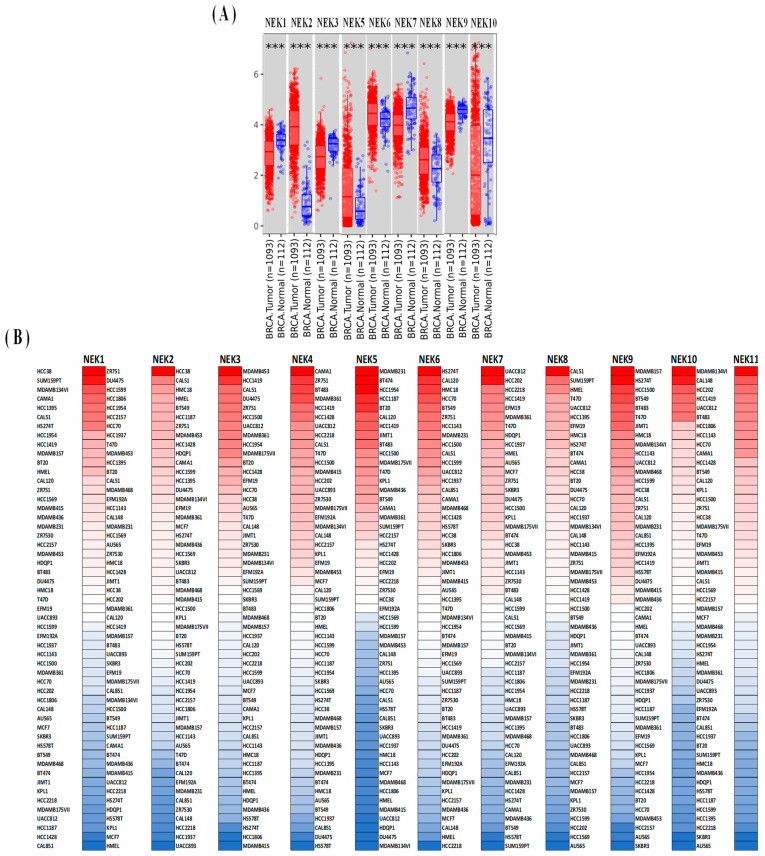
Expression of the *NEK* gene family in breast cancer. (**A**) Box plot of the *NEK* gene family’s transcripts in normal and breast cancer tissues in the TIMER database. Statistical significance was calculated using the Wilcoxon test, *** *p* < 0.001. (**B**) Heatmap of the *NEK* gene family’s expression levels in breast cancer cell lines (CCLE). We used mRNA expression values from the CCLE database, and then displayed them by their ranking. In CCLE, red represents overexpression (top column) and blue indicates under-expression (bottom column).

**Figure 3:**
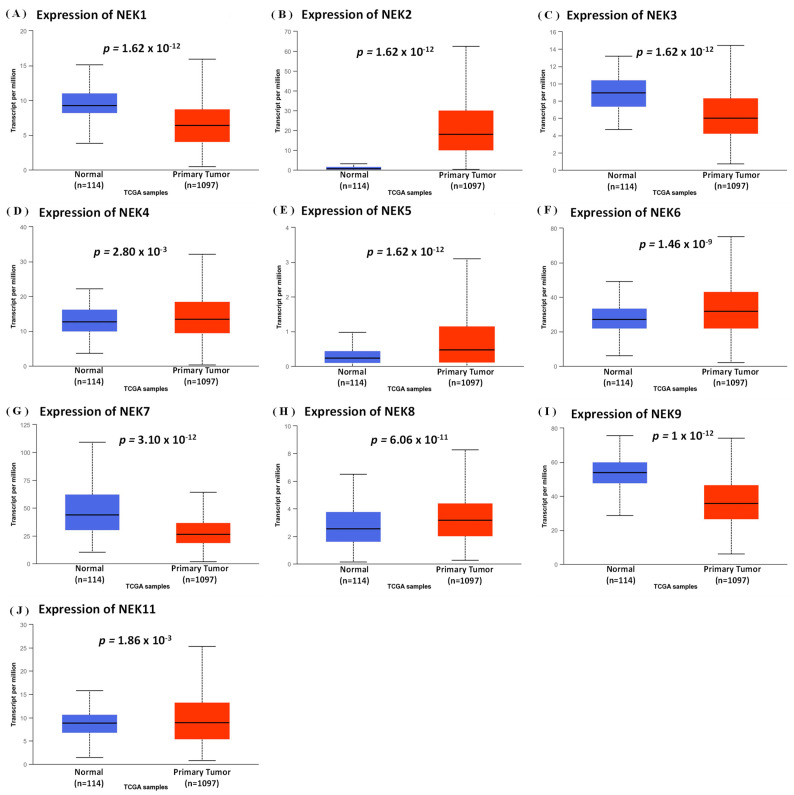
Expression of the *NEK* gene family in breast cancer (UALCAN Analysis). (**A**–**J**) Box plot of *NEK* gene family transcripts in normal and primary tumor (BRCA) tissues. The box plot shows comparisons of the expressions of TCGA data from the *NEK* gene family in breast cancer, between normal samples (*n* = 114) and primary tumors (*n* = 1097). Statistical significance is represented by *p* < 0.05.

**Figure 4:**
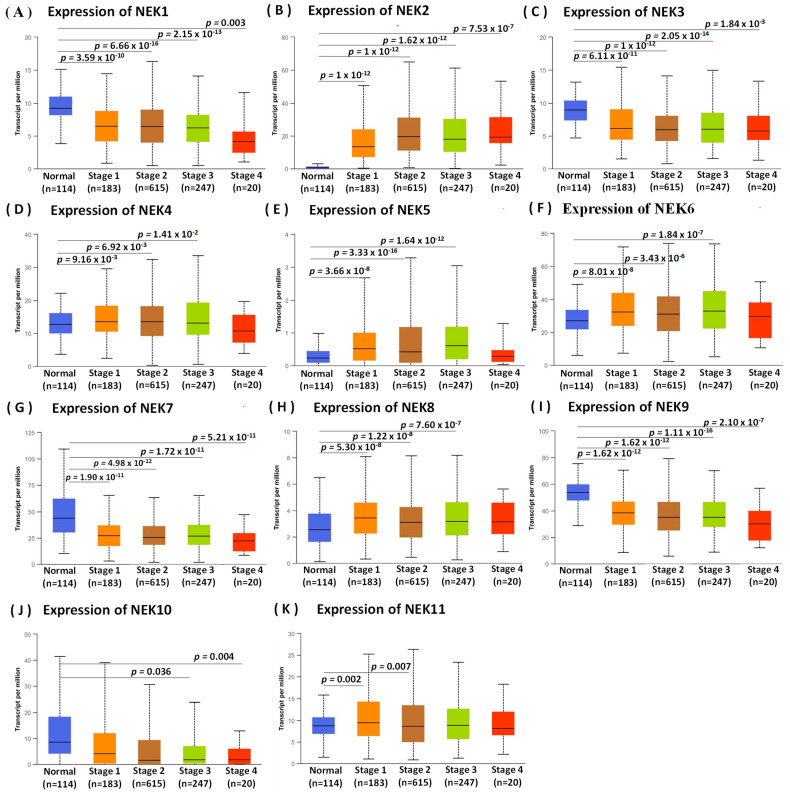
Expression transcript levels of the NEK gene family in normal tissues with individual cancer stages or clinicopathology (UALCAN analysis). (**A**–**K**) Box plots of the NEK gene family transcripts in normal tissues and various tumor stages (breast cancer, BRCA). An independent *t*-test was used to calculate *p* values. Statistical significance was indicated by *p* < 0.05.

In this study, we used TIMER, CCLE, and UALCAN databases (Figure 2, Figure 3 and Figure 4) to reveal the transcriptional expressions of 11 genes of the *NEK* family. We determined the distributions of gene expression levels using the TIMER database, which are displayed in a box plot. This study found significant differences between normal and breast cancer tissues, and we determined that differences in *NEK1/2/3/5/6/7/8/9/10* expressions were statistically significant at *p* < 0.001 (Figure 2A). In addition, we also studied expression levels of NEK gene family members in breast cancer cell lines using the CCLE database (Figure 2B).

Next, we studied mRNA expression patterns of the *NEK* family that differed between normal and breast cancer tissues using the UALCAN database, and also reflected clinical parameters such as tumor stage (Figure 3 and Figure 4). Differences in expressions of *NEK1/2/3/4/5/6/7/8/9/11* were statistically significant (*p* < 0.05) between normal and breast cancer tissues (Figure 3A–J). Furthermore, we found that *NEK1/2/3/7/9/10* mRNA expressions had a trend with higher statistical significance in more advanced tumors based on tumor stage indicators (Figure 4A–K).

### 3.2. Prognostic Value of the NEK Family Members in Breast Cancer

**Figure 5:**
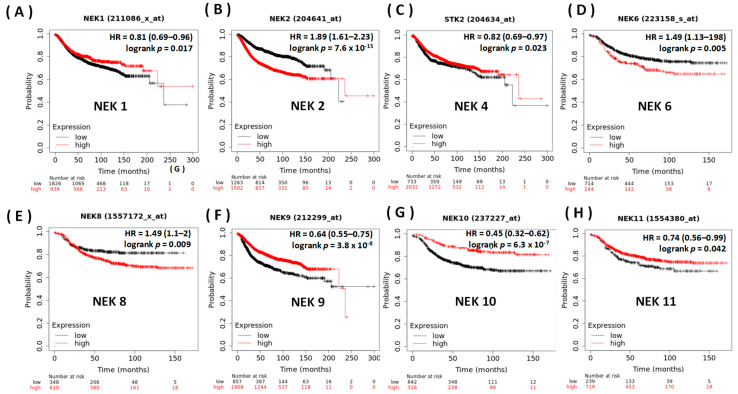
Survival curve of the *NEK* gene family on distant metastasis-free survival (DMFS) in breast cancer patients (Kaplan-Meier plotter). The hazard ratio (HR) is a relative prognostic measure of patients with breast cancer. logrank *p* was used to determine the level of prognostic significance of patients with breast cancer. Furthermore, the logrank *p* < 0.05 was interpreted as a significant difference in the prognostic expression of patients with breast cancer.

We analyzed a breast cancer database with the KM plotter (Figure 5), to unveil the prognostic significance of values of overexpression of NEK family genes on DMFS in breast cancer patients. The KM curve investigation and log-rank test revealed higher expression levels of *NEK1/2/4/6/8/9/10/11* mRNA, and these were significantly correlated with a poor DMFS. As to DMFS, we concluded that most *NEK* genes were significantly correlated with the prognosis of breast cancer patients: *NEK1* (HR = 0.81, 95% CI = 0.69~0.96, *p* = 0.049), *NEK2* (HR = 1.89, 95% CI = 1.61~2.23, *p* = 7 × 10^−15^), *NEK4* (HR = 0.82, 95% CI = 0.69~0.97, *p* = 0.023), *NEK6* (HR = 1.49, 95% CI = 1.13~1.98, *p* = 0.0048), *NEK8* (HR = 1.49, 95% CI = 1.1~2, *p* = 0.0087), *NEK9* (HR = 0.64, 95% CI = 0.55~0.75, *p* = 0.049), *NEK10* (H = 0.45, 95% CI = 0.32~0.62, *p* = 7 × 10^−7^), and *NEK11* (HR = 0.74, 95% CI = 0.56~0.99, *p* = 0.042).

### 3.3. DNA Methylation Analysis of the NEK Family Members in Breast Cancer

DNA methylation is an epigenetic alteration that plays a role in the development of several cancers [77]. DNA methyltransferases on CpG island methylation are transcription factors in the suppression or promotion of cell growth and it is a reversible process [78]. We present heatmap and prognostic value of DNA methylation clustering the expression levels of the NEK gene family in breast cancer (Figure S1 and Table S1 in Supplementary). DNA methylation expression levels concluded that cg17931972 from NEK2 and cg14289738 from NEK6 had the highest DNA methylation levels and significant prognostic value (likelihood ratio (LR) test *p*-value < 0.05) in breast cancer.

Collectively, the results of several integrative analyses such as TIMER, CCLE, UALCAN, survival analysis with the KM plotter, and DNA methylation revealed that several NEK family genes were consistently overexpressed in breast cancer. In the TIMER database, we found that NEK1/2/3/5/6/7/8/9/10 had significant *p* values < 0.001, and in the CCLE analysis, we also found overexpression levels of NEK gene family members in breast cancer cell lines. In the UALCAN analysis, we discovered that NEK1/2/3/4/5/6/7/8/9/11 were statistically significantly overexpressed in breast cancer compared to normal tissues. Furthermore, we found that NEK1/2/3/7/9/10 were overexpressed in terms of clinicopathological indicators and had higher statistical significance in more advanced tumors. We found a significant result in the survival analysis, which revealed that NEK2/6/8 had high HRs and overexpression prognostic significance in DMFS in breast cancer patients. DNA methylation analysis also concluded that NEK2/6 had the highest level of DNA methylation and a significant prognostic value (likelihood ratio (LR) test *p*-value < 0.05) in breast cancer. Therefore, this study further explored the NEK2 gene by investigating MFs, CCs, and BPs using GO and KEGG. Enriched pathways in transcriptional data were evaluated by a GSEA. A functional analysis was conducted on various omics data, which calculated *p* values of enrichment across different gene pools in datasets uploaded on the MetaCore platform. Then, this study also investigated the relationship of NEK2 with miRNA-regulated networks. Finally, we also studied the correlation of NEK2 transcriptional levels with immune infiltration.

### 3.4. Regulated Networks of NEK2 Gene Expressions in Breast Cancer

**Figure 6:**
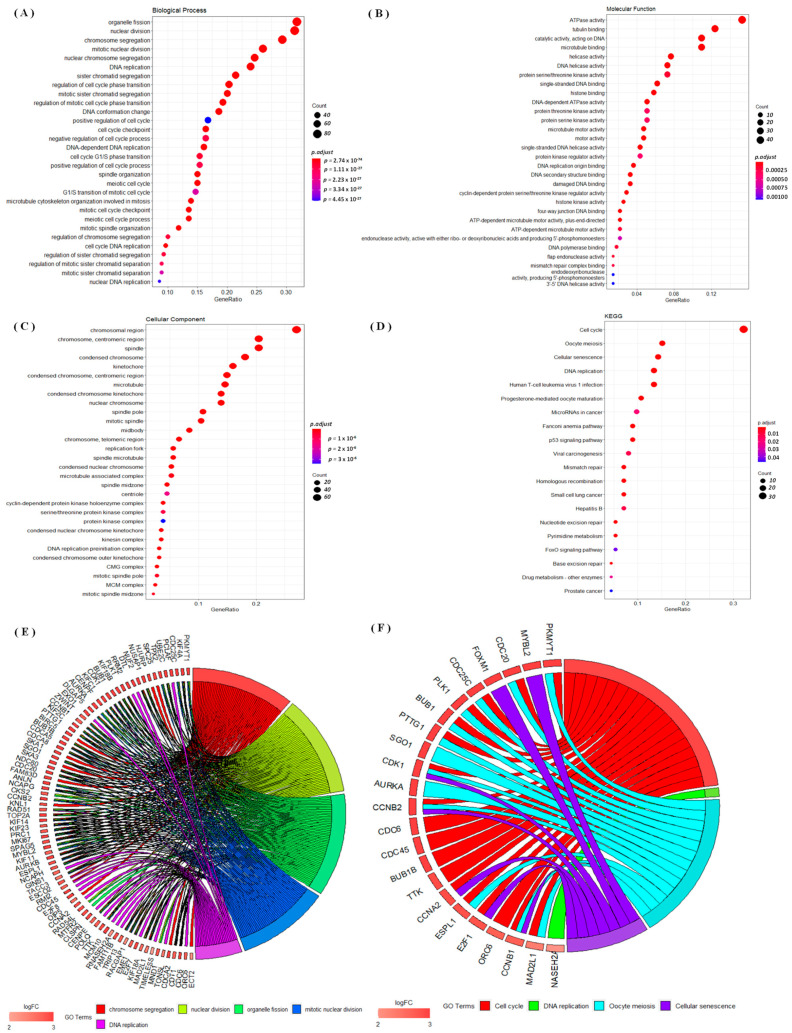
Gene ontology (GO) analysis based on genes co-expressed with *NEK2* from the associated METABRIC and TCGA datasets. (**A**) Dot plot of biological processes. (**B**) Dot plot of cellular components. (**C**) Dot plot of molecular functions. (**D**) Dotplot of KEGG. The dot size is determined by the count of enriched genes in the pathway, and the color of the dots represents the pathway enrichment’s significance. We used the “clusterProfiler” package in R/Bioconductor to perform the GO analyses of dot plots. (**E**) Chord plot of relationships between genes and GO terms of biological processes. (**F**) Chord plot of relationships between genes and GO terms of KEGG.

This study performed a GO analysis based on genes co-expressed by *NEK2* from associated METABRIC and TCGA datasets (Figure 6). This analysis investigated BPs, CCs, and MFs that were affected under the conditions studied. We found that genes co-expressed with NEK2 from the METABRIC and TCGA datasets were involved in organelle fission, nuclear division, and chromosome segregation in BPs (Figure 6A); ATPase activity, tubulin binding, and catalytic activity-acting on DNA in MFs (Figure 6B); chromosomal region, chromosome-centromeric region, and the spindle in CCs (Figure 6C); and cell cycle, oocyte meiosis, and cellular senescence in KEGG (Figure 6D). We also studied the differential involvement of gene expressions (DEGs) with GO terms of BPs and KEGG displayed in chord plots, and we found it to be mostly upregulated (Figure 6E,F).

**Figure 7:**
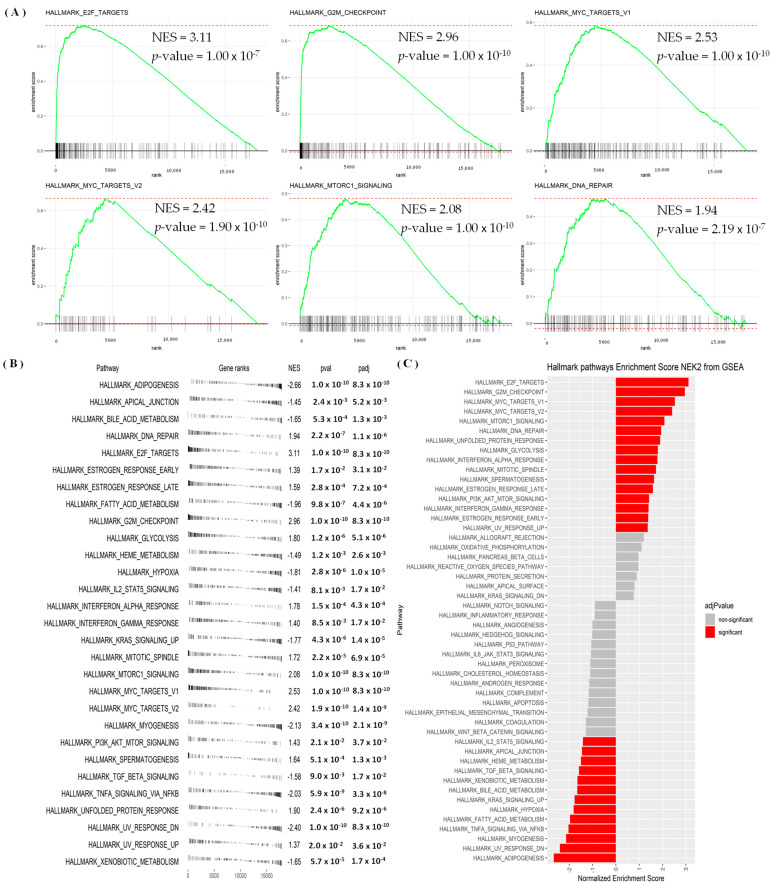
(**A**–**C**) Hallmark signaling pathway analysis of *NEK2* in breast cancer. We used median expression of the *NEK2* gene (high and low expression) and then performed a differential analysis using the algorithm in the “DESeq2” package in R/Bioconductor. Furthermore, results of the differential analysis were used as input for the gene set enrichment analysis (GSEA) with the Hallmark database, and computationally used the “fgsea” package in R/Bioconductor. Results of the analysis show significant values of gene classes in the Hallmark database. The level of statistical significance is shown through the *p*-value, and the normalized enrichment score (NES) reflects the rank of gene classes in the database.

**Figure 8:**
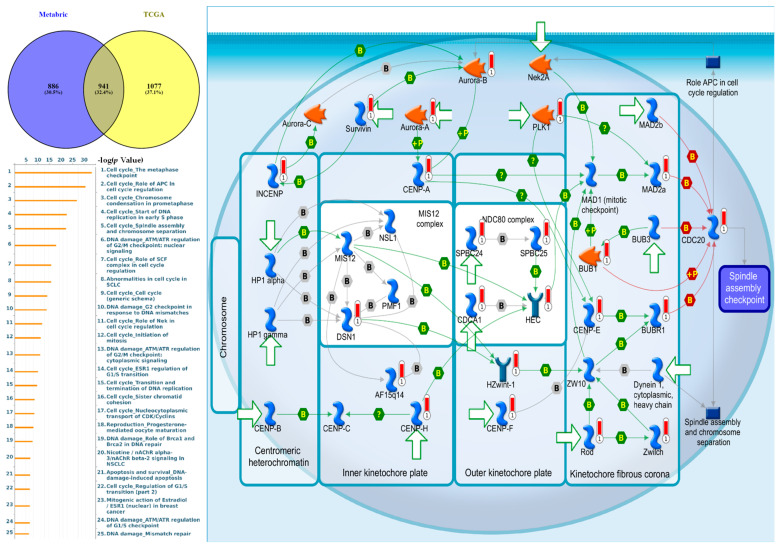
Expression of the NEK2 signaling pathway in breast cancer (MetaCore). We used the MetaCore platform to analyze genes co-expressed with NEK2 from the associated METABRIC and TCGA datasets, and we found that “Cell cycle_The metaphase checkpoint” was correlated with breast cancer development (with *p* < 0.05 set as the cutoff value).

GSEA results revealed that hallmark analysis signaling pathways significantly involved E2F targets, G2M checkpoint, MYC targets, MTORC1 signaling, and DNA repair (Figure 7A–C, Table S2 in Supplementary). We used the MetaCore platform to reveal the functions, enrichment pathways, and network analyses of the *NEK2* gene in breast cancer. *NEK2* co-expressed genes from the METABRIC and TCGA breast cancer datasets were analyzed by MetaCore and revealed that *NEK2* co-expressed genes were involved in cell cycle process including “The metaphase checkpoint”, “Role of APC in cell cycle regulation”, “Chromosome condensation in prometaphase”, “Start of DNA replication in early S phase”, and “Spindle assembly and chromosome separation” in breast cancer development (Figure 8, Table S3 in Supplementary). Furthermore, analysis of miRNA-regulated networks with *NEK2* also suggested that hsa-miR-1236-3p, hsa-miR-4264, hsa-miR-486-5p, hsa-miR-155-3p, and hsa-miR-6839-3p are also regulated breast cancer development (Figure S2 in Supplementary).

### 3.5. Levels of Immune Infiltration in Breast Cancer Were Linked to Expression of NEK2

**Figure 9:**
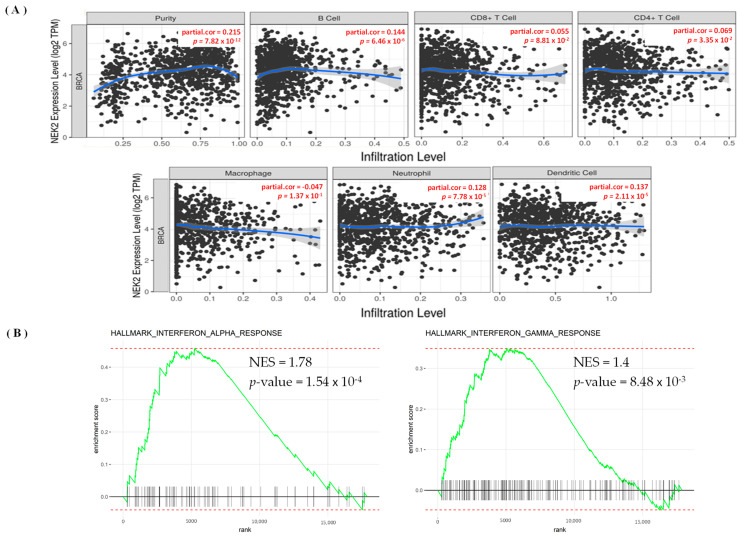
(**A**) Scatterplot of correlations among expressions of the *NEK2* gene and levels of immune infiltration in breast cancer. Correlation of *NEK2* with levels of immune infiltration (purity, B cells, CD8^+^ T cells, CD4^+^ T cells, macrophages, neutrophils, and dendritic cells). The correlation measurement is indicated by the partial correlation value using Spearman’s partial rho and the statistical significance of the *p* value. (**B**) GSEA analysis suggested that *NEK2* expression could regulate the interferon alpha and gamma signaling in breast cancer.

The TIMER database was used to explore the immunological microenvironment and identified correlations between levels of immune infiltration and expressions of the *NEK2* gene in breast cancer (Figure 9). NEK2 expression was significantly positively linked with immune infiltration of B cells (r = 0.144, *p* = 6.46 × 10^−6^), purity (r = 0.215, *p* = 7.82 × 10^−12^), CD4^+^ T cells (r = 0.069, *p* = 0.033), neutrophils (r = 0.128, *p* = 7.78 × 10^−5^), and DCs (r = 0.137, *p* = 2.11 × 10^−5^) (Figure 9A). Subsequently, we explored more deeply using several immune level algorithms of B cells and CD4^+^ T cells. Previous studies suggested that B cells and CD4^+^ T cells have essential functions in developing immune-based therapies in all disease subtypes [79,80,81]. This study found that NEK2 gene expression was positively and significantly correlated with B cell, such as the naïve XCELL (r = 0.109, *p* = 0.001), QUANTISEQ (r = 0.180, *p* = 0.000), XCELL (r = 0.202, *p* = 0.000), and Class-switched memory XCELL (r = 0.101, *p* = 0.001) (Figure S3 and Table S4 in Supplementary). Furthermore, in terms of CD4^+^ T immune cells, we found that NEK2 gene expression was positively and significantly correlated with T cell CD4^+^ memory, such as the activated CIBERSORT (r = 0.2223, *p* = 0.000), activated CIBERSORT-ABS (r = 0.2224, *p* = 0.0000), resting CIBERSORT-ABS (r = 0.1065, *p* = 0.0008), XCELL (r = 0.2963, *p* = 0.000), Th1 XCELL (r = 0.1474 *p* = 0.000), and Th2 XCELL (r = 0.6766, *p* = 0.000) (Figure S4 and Table S5 in Supplementary). We also found associations between BPs of *NEK2* in the GSEA analysis and immune categories. Expression of NEK2 correlated with hallmark interferon alpha and gamma responses (*p* = 1.54 × 10^−4^, NES = 1.78) and (*p* = 8.48 × 10^−3^, NES = 1.4), respectively (Figure 7B and Table S2 in Supplementary).

## 4. Discussion

According to previous studies, NEK dysregulation was linked to the occurrence and progression of several cancers [82,83]. We know that NEKs regulation processes of cell death and senescence in addition to tumor cell proliferation and differentiation [82,84,85]. Although the involvement of NEKs in the incidence and survival of some malignancies was reported, the holistic approach to explore the roles of distinct NEKs in the development of breast cancer still remains largely unexplored. Therefore, this is the first study to use bioinformatics and integrate data mining of biological databases to investigate transcription levels, and biological functions of distinct NEK family members in breast cancer, as well as their associations with prognosis and immunological infiltration in breast cancer patients. In further analysis, this study also investigated the effects of gene expressions on immune cell infiltration. Molecular and cellular factors of immune cell infiltration play essential roles in cancer BPs and are particularly useful in predicting OS and guiding treatment for patients with breast cancer [86,87,88,89,90]. We also investigated the interaction of a potential network of genes from the *NEK* family with miRNA. miRNA is a major post-transcriptional gene expression regulator known to play an important role in regulating cancer development [72,91,92].

Our comprehensive study of 11 members of the current, exploratory *NEK* gene family reveals that *NEK2/6/8* were closely related to the development of breast cancer in humans. We found a significant result in the survival analysis, which revealed that *NEK2/6/8* have high HRs and overexpression prognostic significance in DMFS in breast cancer patients. Meanwhile, a previous study revealed that *NEK2* has distinctly essential roles as a tumor-suppressor gene in different cancers, such as lung adenocarcinoma [75], ovarian cancer [76], hepatocellular carcinoma [93], and breast cancer [94]. The high expression of NEK2 has also been identified in prostate cancer [95]. Moreover, NEK6 also plays a role as a tumor-suppressor gene in different cancers, including thyroid cancer [96], gastric cancer [97], hepatic cell cancer [98], and breast cancer [99]. Furthermore, NEK8 is known to play a role in gastric cancer cells [100] and breast cancer [25], while NEK11 is known to play a role in ovarian cancer [101]. To reveal the prognostic potential of *NEK2* and its relationship with other prognostics, we used integrated data analysis with high-throughput technology and several bioinformatics tools.

This study found that the *NEK2* gene was significantly overexpressed in human breast cancer tissues compared to normal tissues through the TIMER and UALCAN exploration. Based on the clinicopathological characteristics of the tumor stage, we discovered that *NEK2* mRNA expression tended to be significantly higher in more advanced tumor stages, whereas *NEK6/8/11* mRNA expressions were only found in tumor stages 1, 2, and 3. In DNA methylation analysis, this study found that the prognostic value of NEK2/6 in a single CpG was significant in breast cancer development. We found prognostic significance of DNA methylation expression levels in cg17931972 from NEK2 and cg14289738 from NEK6. As a result of our investigation, we found that NEK2 consistently has a poor prognosis in breast cancer. Therefore, we thoroughly investigated the biological processes of NEK2 and the regulation of NEK2 with miRNA.

Results of the GO analysis revealed a significant presence of *NEK* gene family members in the development of breast cancer. The GO analysis of genes co-expressed with *NEK2* from the METABRIC and TCGA datasets showed that almost all of them were associated with cell division and DNA replication in BPs [102,103], ATPase activity in MFs [104,105,106,107], chromosomal regions in CCs [108], and cell cycles and oocyte meiosis in KEGG [109,110,111]. The study revealed the involvement of DEGs with GO terms of BPs and KEGG. PKMYT1, KIF4A, and CDC25C were associated with GO in terms of BPs; previous studies also revealed the overexpression of these genes in breast cancer [112,113,114]. PKMYT1, MYBL2, and CDC20 participate in GO terms of the most upregulated KEGG processes and are involved in the development of breast cancer [113,115,116,117]. The GSEA result also revealed that the high expression of NEK2 groups in the TCGA breast cancer database were significantly correlated with G2M checkpoint, E2F, MYC targets signaling pathways [118,119,120]. An investigative analysis of the network interaction between NEK2 and miRNA revealed hsa-miR-1236-3p, hsa-miR-4264, hsa-miR-486-5p, hsa-miR-155-3p, and hsa-miR-6839-3p are co-expressed for breast cancer development. In a recent study, the miRNA hsa-miR-1236-3p was identified as having overexpression at the TNM stage and metastases of colon cancer [121]. Meanwhile, hsa-miR-486-5p has been identified as a prognostic biomarker and therapeutic target in lung cancer [122]. The results of miRNA investigations in this study are also consistent with previous studies that found the involvement of hsa-miR-155-3p in breast cancer [123].

The MetaCore results of this study revealed a high correlation between NEK2 and the cell cycle and metaphase checkpoint pathway in breast cancer development [124,125,126]. This pathway was associated with several genes, including *CDCA1, CDC20, MAD2a, PLK1, Aurora-A, BUB1*, and *BUBR1* (Supplementary Table S3, Figure 8). Meanwhile, PLK1 overexpressed in the liver, lung, stomach, and epidermis [127], BUBR1 (also known as homologue beta) is overexpressed in colorectal cancer, lung, pancreatic tumors, and T-cell lymphoma [128,129]. Cell cycle regulation of protein kinases plays a significant and potentially exciting role in cancer therapeutics [130]. In cell cycle kinases in human cancers, previous studies have revealed that Aurora-A is overexpressed in several human tumors, including breast, colorectal, and bladder cancers [131,132].

A previous study demonstrated the critical roles of cell-cycle checkpoint processes and DNA repair in cancer development because of their respective functions in regulating genome stability and cell development [133]. Furthermore, cell-cycle checkpoints have the potential to significantly improve cancer treatments [118]. Our data also revealed that NEK family genes were correlated with cell cycle regulation. However, since data of the study were acquired only by performing integrated bioinformatics analyses, further in vitro and in vivo experiments should be designed, because these current findings provide clearer insights into the functions of NEK family genes in breast cancer. This will definitely improve the treatment and management of breast cancer patients [134,135,136,137,138].

Finally, we identified NEK2 as a potential biomarker of immune cells in breast cancer tissues. We discovered the correlation between the NEK2 gene’s expression and immunological levels of B cells, CD4^+^ T cells, neutrophils, and DCs. These findings are consistent with current research, revealing that B cells provide a potential target for cancer intervention [139,140]. Previous studies have also shown that CD4^+^ T cells significantly inhibit tumor development [141], neutrophils [142,143,144], and DCs [145,146,147,148], and our study also revealed correlations of the NEK2 gene with several immune infiltration rate algorithms in breast cancer tissues. We found high correlations of NEK2 gene expression with B cell and T cell, CD4^+^ Th2 cell. Another interesting finding in this study is the link between NEK2 BPs and immunological categories in the GSEA analysis. We found that NEK2 was correlated with hallmark interferon-alpha and gamma responses (*p* = 1.54 × 10^−4^, NES = 1.78) and (*p* = 8.48 × 10^−3^, NES = 1.4), respectively (Supplementary Table S2, Figure 7B). Interestingly, these findings are consistent with previous studies, which suggested that interferon regulators play an essential role in developing anti-tumor immunity and post-chemotherapy metastasis-free survival of triple-negative breast cancer (TNBC) [149]. In addition, previous studies have also reported a correlation between interferon regulation and T cell signature [150,151]. Therefore, NEK2 has the potential as a prognostic biomarker for immune infiltration in breast cancer development.

## 5. Conclusions

Our study revealed that among all members of the NEK family, NEK2 is overexpressed in breast cancer patients and is associated with a poor prognosis in breast cancer. On the enrichment pathway, we also discovered an important role of NEK2 on the cell cycle and metaphase checkpoint regulation. In conclusion, NEK2 may have potential value as a prognostic and immune infiltration marker for breast cancer development.

## Figures and Tables

**Table 1:** Basic Characteristics of the NEK Genes Family.

Approved Symbol	HGNC ID	Gene ID	Aliases	Location on Chromosome
NEK1	7744	4750	NY-REN-55; KIAA1901	4q33
NEK2	7745	4751	NLK1; PPP1R111; RP67	1q32.3
NEK3	7746	4752	HSPK36	13q14.3
NEK4	11399	6787	Pp12301; NRK2; STK2	3p21.1
NEK5	7748	341676	EC 2.7.11.1	13q14.3
NEK6	7749	10783	SID6-1512	9q33.3
NEK7	13386	140609	EC 2.7.11.1	1q31.3
NEK8	13387	284086	NPHP9	17q11.2
NEK9	18591	91754	NERCC; DKFZp434D0935	14q24.3
NEK10	18592	152110	FLJ32685; CILD44	3p24.1
NEK11	18593	79858	FLJ23495; EC 2.7.11.1	3q22.1

## Data Availability

The datasets used and/or analyzed during the current study are available from the corresponding author upon reasonable request.

## Supplementary Materials

The following are available online at https://www.mdpi.com/article/10.3390/jpm11111089/s1, Figure S1: Heatmap of DNA methylation expression levels of the NEK gene family in breast cancer by MethSurv platform. cg02998883, cg05110629, cg26722769 of NEK1; cg15831905, cg17931972 of NEK2; cg19524009, cg22056112 of NEK3; cg02636488 of NEK4; cg15721359, cg18615369 of NEK5; cg14536906, cg13866149, cg13974765, cg14289738 of NEK6; cg04223956, cg09372617 of NEK7; cg17742559 of NEK8; cg04246305 of NEK9; cg09642369, cg17918906 of NEK10; cg01378599, cg06239593 of NEK11 displays the highest level of DNA methylation in breast cancer. Table S1: Prognostic Value of Single CpG of the NEK gene family in breast cancer by MethSurv platform. The threshold of significance was LR Test *p*-value <0.05. A significant expression pattern was found in NEK2/6 between low and high risk groups for breast cancer. Table S2: Hallmark signaling pathway analysis of NEK2 in BRCA (GSEA Analysis). Table S3: Pathway analysis of genes coexpressed NEK2 from public breast cancer databases using the MetaCore database (with *p*-value < 0.05 set as the cutoff value). Figure S2: Analysis of micro (mi)RNA networks with NEK2 in breast cancer. We used the miRWalk database to identify associations with NEK2, and then network regulation was analyzed by Ingenuity Pathway Analysis (IPA). hsa-miR-1236-3p, hsa-miR-4264, hsa-miR-486-5p, hsa-miR-155-3p, and hsa-miR-6839-3p are co-expressed for breast cancer development. Table S4: Correlation of NEK2 expression with the level of immune infiltration of B cells in BRCA (Analysis of TIMER database). The data shows the partial correlation value, and the level of statistical significance is shown with *p*-value and adjusted *p*-value. The threshold of significance was *p*-value < 0.05. Figure S3: Heatmap of NEK2 expression with the level of immune infiltration of B cells in different types of cancer (Analysis of TIMER database). The partial correlation coefficient shows the size of the correlation, and the *p*-value indicates the level of statistical significance. The threshold of significance was *p*-value < 0.05. Table S5: Correlation of NEK2 expression with the level of immune infiltration of CD4+ T Cells in BRCA (Analysis of TIMER database). rho shows the partial correlation value, and the level of statistical significance is shown with p-value and adjusted *p*-value. The threshold of significance was *p*-value < 0.05. Figure S4: Heatmap of NEK2 expression with the level of immune infiltration of CD4+ T Cells in different types of cancer (Analysis of TIMER database). The partial correlation coefficient shows the size of the correlation, and the *p*-value indicates the level of statistical significance. The threshold of significance was *p*-value < 0.05.
