# Supplementary material for: Potential Prognostic Biomarkers of NIMA (Never in Mitosis, Gene A)-Related Kinase (NEK) Family Members in Breast Cancer
Source: J Pers Med. 2021 Oct 26;11(11):1089. doi: 10.3390/jpm11111089 (PMC8625415; doi:10.3390/jpm11111089)
Supplement: Supplementary file 1 [file jpm-11-01089-s001.zip › jpm-1417532-supplementary.pdf]

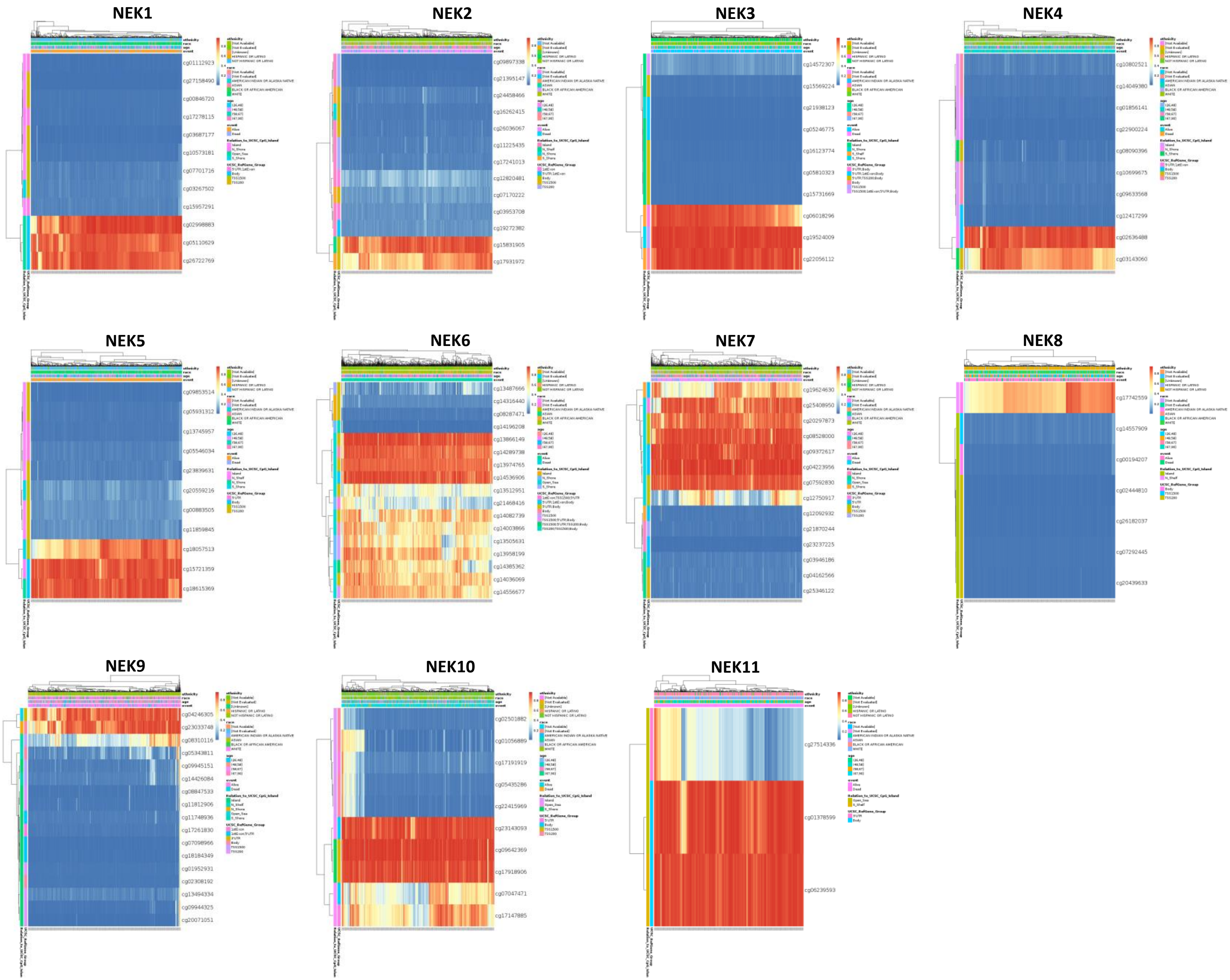
NEK1
NEK2
NEK3
NEK4
NEK5
NEK6
NEK7
NEK8
NEK9
NEK10
NEK11

Figure S1. Heatmap of DNA methylation expression levels of the NEK gene family in breast cancer by MethSurv platform. cg02998883, cg05110629, cg26722769 of NEK1; cg15831905, cg17931972 of NEK2; cg19524009, cg22056112 of NEK3; cg02636488 of NEK4; cg15721359, cg18615369 of NEK5; cg14536906, cg13866149, cg13974765, cg14289738 of NEK6; cg04223956, cg09372617 of NEK7; cg17742559 of NEK8; cg04246305 of NEK9; cg09642369, cg17918906 of NEK10; cg01378599, cg06239593 of NEK11 displays the highest level of DNA methylation in breast cancer.

Table S1: Prognostic Value of Single CpG of the NEK gene family in breast cancer by MethSurv platform. The threshold of significance was LR Test p-value <0.05. A significant expression pattern was found in NEK2/6 between low and high risk groups for breast cancer.

Gene-CpG	HR	LR Test p-value
NEK1-Body-Open_Sea-cg02998883	1.216	0.33
NEK1-Body-Open_Sea-cg05110629	0.816	0.3
NEK1-Body-Open_Sea-cg26722769	0.751	0.16
NEK2-Body-N_Shelf-cg15831905	0.971	0.88
NEK2-TSS1500-S_Shore-cg17931972	0.558	0.0036*
NEK3-TSS1500-S_Shore-cg19524009	1.219	0.32
NEK3-Body-S_Shelf-cg22056112	0.77	0.19
NEK4-Body-N_Shore-cg02636488	1.073	0.72
NEK5-Body-N_Shelf-cg15721359	0.847	0.4
NEK5-Body-N_Shore-cg18615369	0.845	0.4
NEK6-5'UTR;1stExon;Body-Open_Sea-cg14536906	0.879	0.51
NEK6-5'UTR;Body-Open_Sea-cg13866149	0.963	0.85
NEK6-5'UTR;Body-Open_Sea-cg13974765	0.695	0.068
NEK6-Body-Open_Sea-cg14289738	0.56	0.0042*
NEK7-5'UTR-Open_Sea-cg04223956	0.976	0.9
NEK7-5'UTR-Open_Sea-cg09372617	1.15	0.48
NEK8-Body-N_Shelf-cg17742559	0.858	0.44
NEK9-3'UTR-Open_Sea-cg04246305	0.699	0.07
NEK10-TSS1500-S_Shore-cg09642369	0.749	0.14
NEK10-TSS1500-S_Shore-cg17918906	0.971	0.88
NEK11-Body-Open_Sea-cg01378599	1.056	0.78
NEK11-Body-S_Shelf-cg06239593	0.799	0.26

Table S2: Hallmark signaling pathway analysis of NEK2 in BRCA (GSEA Analysis, Supplementary table S2, Figure 7).

Pathway	pval	padj	log2err	ES	NES
HALLMARK_E2F_TARGETS	1.00E-10	8.33E-10		0.71994	3.11495
HALLMARK_G2M_CHECKPOINT	1.00E-10	8.33E-10		0.683988	2.959398
HALLMARK_MYC_TARGETS_V1	1.00E-10	8.33E-10		0.585011	2.531697
HALLMARK_MYC_TARGETS_V2	1.90E-10	1.35E-09	0.826657	0.664633	2.415173
HALLMARK_MTORC1_SIGNALING	1.00E-10	8.33E-10		0.481279	2.082339
HALLMARK_DNA_REPAIR	2.19E-07	1.09E-06	0.690132	0.46777	1.943888
HALLMARK_UNFOLDED_PROTEIN_RESPONSE	2.40E-06	9.22E-06	0.627257	0.473169	1.898675
HALLMARK_GLYCOLYSIS	1.22E-06	5.09E-06	0.643552	0.416807	1.803391
HALLMARK_INTERFERON_ALPHA_RESPONSE	0.000154	0.000427	0.518848	0.455404	1.783309
HALLMARK_MITOTIC_SPINDLE	2.20E-05	6.87E-05	0.57561	0.397629	1.720994
HALLMARK_SPERMATOGENESIS	0.000511	0.001253	0.477271	0.398886	1.639428
HALLMARK_ESTROGEN_RESPONSE_LATE	0.000275	0.000725	0.498493	0.367052	1.588115
HALLMARK_PI3K_AKT_MTOR_SIGNALING	0.021276	0.036682	0.352488	0.358774	1.427884
HALLMARK_INTERFERON_GAMMA_RESPONSE	0.008478	0.016956	0.38073	0.324046	1.402341
HALLMARK_ESTROGEN_RESPONSE_EARLY	0.016549	0.030646	0.352488	0.320301	1.386305
HALLMARK_UV_RESPONSE_UP	0.020007	0.035726	0.352488	0.327529	1.369654
HALLMARK_ALLOGRAFT_REJECTION	0.110682	0.172941	0.149208	0.275883	1.193914
HALLMARK_OXIDATIVE_PHOSPHORYLATION	0.240669	0.325229	0.095288	0.256042	1.10805
HALLMARK_PANCREAS_BETA_CELLS	0.50234	0.558156	0.066284	0.290154	0.976768
HALLMARK_REACTIVE_OXYGEN_SPECIES_PATHWAY	0.490798	0.557724	0.066589	0.279271	0.97531
HALLMARK_PROTEIN_SECRETION	0.678273	0.721567	0.046882	0.228996	0.895558
HALLMARK_APICAL_SURFACE	0.806854	0.82332	0.044064	0.235104	0.801411
HALLMARK_KRAS_SIGNALING_DN	0.962773	0.962773	0.026395	0.177094	0.766229
HALLMARK_NOTCH_SIGNALING	0.634921	0.690131	0.081303	-0.254	-0.89576
HALLMARK_INFLAMMATORY_RESPONSE	0.748879	0.780082	0.10208	-0.18367	-0.90667
HALLMARK_ANGIOGENESIS	0.43454	0.50528	0.106323	-0.27918	-1.00469
HALLMARK_HEDGEHOG_SIGNALING	0.383152	0.456134	0.112843	-0.28342	-1.01703
HALLMARK_P53_PATHWAY	0.327273	0.409091	0.164406	-0.21337	-1.05046

**HALLMARK_IL6_JAK_STAT3_SIGNALING**	0.347079	0.423267	0.13649	-0.24641	-1.06307
**HALLMARK_PEROXISOME**	0.279851	0.358783	0.160801	-0.24548	-1.09361
**HALLMARK_CHOLESTEROL_HOMEOSTASIS**	0.270548	0.355984	0.156312	-0.26434	-1.10124
**HALLMARK_ANDROGEN_RESPONSE**	0.197183	0.273865	0.188204	-0.25668	-1.14047
**HALLMARK_COMPLEMENT**	0.15521	0.221729	0.249247	-0.23571	-1.16046
**HALLMARK_APOPTOSIS**	0.142276	0.20923	0.249247	-0.24794	-1.1832
**HALLMARK_EPITHELIAL_MESENCHYMAL_TRANSITION**	0.061505	0.099202	0.321776	-0.24459	-1.21122
**HALLMARK_COAGULATION**	0.040394	0.067324	0.321776	-0.2757	-1.28536
**HALLMARK_WNT_BETA_CATENIN_SIGNALING**	0.119241	0.180668	0.213928	-0.34332	-1.2893
**HALLMARK_IL2_STAT5_SIGNALING**	0.008083	0.016839	0.38073	-0.2864	-1.41003
**HALLMARK_APICAL_JUNCTION**	0.002407	0.005233	0.431708	-0.29583	-1.45268
**HALLMARK_HEME_METABOLISM**	0.001156	0.002627	0.45506	-0.30426	-1.4941
**HALLMARK_TGF_BETA_SIGNALING**	0.008964	0.017238	0.38073	-0.39871	-1.5794
**HALLMARK_XENOBIOTIC_METABOLISM**	5.68E-05	0.000167	0.557332	-0.33553	-1.64767
**HALLMARK_BILE_ACID_METABOLISM**	0.000526	0.001253	0.477271	-0.37067	-1.65469
**HALLMARK_KRAS_SIGNALING_UP**	4.30E-06	1.43E-05	0.610527	-0.357	-1.76789
**HALLMARK_HYPOXIA**	2.81E-06	1.00E-05	0.627257	-0.36633	-1.80838
**HALLMARK_FATTY_ACID_METABOLISM**	9.78E-07	4.45E-06	0.643552	-0.41076	-1.96252
**HALLMARK_TNFA_SIGNALING_VIA_NFKB**	5.89E-09	3.27E-08	0.761461	-0.4113	-2.03038
**HALLMARK_MYOGENESIS**	3.39E-10	2.12E-09	0.814036	-0.43359	-2.13463
**HALLMARK_UV_RESPONSE_DN**	1.00E-10	8.33E-10		-0.51101	-2.39865
**HALLMARK_ADIPOGENESIS**	1.00E-10	8.33E-10		-0.53743	-2.66138

Table S3: Pathway analysis of genes coexpressed NEK2 from public breast cancer databases using the MetaCore database (with p – value <0.05 set as the cutoff value) (Supplementary Table S3, Figure 8).

#	Maps	pValue	Network Objects from Active Data
**1**	Cell cycle_The metaphase checkpoint	6.790E-34	INCENP, Aurora-B, BUB1, HEC, SPBC25, CDCA1, CENP-A, CDC20, HZwint-1, Rod, CENP-F, SPBC24, DSN1, Zwilch, MAD2a, Survivin, CENP-H, CENP-E, AF15q14, Aurora-A, PLK1, BUBR1
**2**	Cell cycle_Role of APC in cell cycle regulation	3.599E-31	Aurora-B, BUB1, CDC25A, CDC18L (CDC6), CDC20, SKP2, Cyclin B, Tome-1, Geminin, MAD2a, Emi1, Securin, ORC1L, Cyclin A, CDK1 (p34), Aurora-A, PLK1, CDK2, CKS1, BUBR1
**3**	Cell cycle_Chromosome condensation in prometaphase	2.439E-27	INCENP, Aurora-B, BRRN1, CAP-H/H2, CAP-G, CAP-E, Cyclin B, Condensin, CAP-C, TOP2, Cyclin A, CNAP1, CAP-G/G2, CDK1 (p34), Aurora-A, CAP-D2/D3
**4**	Cell cycle_Start of DNA replication in early S phase	6.421E-23	Cdt1, CDC18L (CDC6), MCM10, ORC6L, Geminin, ASK (Dbf4), MCM4/6/7 complex, MCM4, CDC7, MCM3, MCM2, ORC1L, Cyclin E, CDK2, E2F1, CDC45L
**5**	Cell cycle_Spindle assembly and chromosome separation	1.233E-22	KNSL1, Aurora-B, HEC, Importin (karyopherin)-alpha, CDC20, TPX2, Tubulin alpha, Cyclin B, CSE1L, MAD2a, Separase, Securin, Ran, CDK1 (p34), Aurora-A, Tubulin (in microtubules)
**6**	DNA damage_ATM/ATR regulation of G2/M checkpoint: nuclear signaling	2.856E-18	CDC25C, GTSE1, WDHD1, CDC18L (CDC6), Cyclin B1, Cyclin B, Chk2, Cyclin B2, TTK, Cyclin A, CDK1 (p34), Claspin, Chk1, PLK1, CDK2
**7**	Cell cycle_Role of SCF complex in cell cycle regulation	3.422E-16	Cdt1, CDC25A, SKP2, Emi1, Cyclin E, CDK1 (p34), Skp2/TrCP/FBXW, Chk1, PLK1, CDK2, CKS1, E2F1
**8**	Abnormalities in cell cycle in SCLC	3.422E-16	Aurora-B, PCNA, Cyclin B1, SKP2, E2F2, Cyclin A, Cyclin E, CDK1 (p34), Cyclin E2, CDK2, CKS1, E2F1
**9**	Cell cycle_Cell cycle (generic schema)	1.878E-14	CDC25C, CDC25A, Cyclin B, E2F2, p107, Cyclin A, Cyclin E, CDK1 (p34), CDK2, E2F1
**10**	DNA damage_G2 checkpoint in response to DNA mismatches	3.081E-14	CDC25C, EXO1, TOPBP1, PCNA, MutSalpha complex, MSH2, Chk2, MSH6, CDK1 (p34), Claspin, Chk1
**11**	Cell cycle_Role of Nek in cell cycle regulation	3.060E-12	HEC, Tubulin beta, Cyclin B1, TPX2, Tubulin alpha, MAD2a, Ran, CDK1 (p34), Aurora-A, Tubulin (in microtubules)

**12**	Cell cycle_Initiation of mitosis	1.347E-11	KNSL1, CDC25C, Lamin B, Cyclin B1, Cyclin B2, FOXM1, Kinase MYT1, CDK1 (p34), PLK1
**13**	DNA damage_ATM/ATR regulation of G2/M checkpoint: cytoplasmic signaling	2.153E-11	CDC25C, Aurora-B, UBE2C, CDC25A, Cyclin B1, BORA, Chk2, CDK1 (p34), Chk1, Aurora-A, PLK1
**14**	Cell cycle_ESR1 regulation of G1/S transition	2.770E-10	Cyclin A2, CDC25A, SKP2, Cyclin A, Cyclin E, Skp2/TrCP/FBXW, CDK2, CKS1, E2F1
**15**	Cell cycle_Transition and termination of DNA replication	5.710E-10	TOP2 alpha, PCNA, MCM2, TOP2, FEN1, Cyclin A, CDK1 (p34), CDK2
**16**	Cell cycle_Sister chromatid cohesion	7.790E-09	PCNA, Cyclin B, DCC1, RFC3, Separase, Securin, CDK1 (p34)
**17**	Cell cycle_Nucleocytoplasmic transport of CDK/Cyclins	8.763E-09	Importin (karyopherin)-alpha, Cyclin B1, Cyclin A, Cyclin E, CDK1 (p34), CDK2
**18**	Reproduction_Progesterone-mediated oocyte maturation	2.429E-08	CDC25C, BUB1, Cyclin B1, CDC20, Kinase MYT1, CDK1 (p34), Aurora-A, PLK1
**19**	DNA damage_Role of Brca1 and Brca2 in DNA repair	6.018E-08	PCNA, FANCD2, BRIP1, MSH2, Chk2, Rad51, MSH6
**20**	Nicotine / nAChR alpha-3/nAChR beta-2 signaling in NSCLC	5.946E-07	CDC25A, CDC18L (CDC6), E2F2, TYSY, Survivin, E2F1
**21**	Apoptosis and survival_DNA-damage-induced apoptosis	7.219E-07	BLM, FANCD2, Chk2, Chk1, E2F1
**22**	Cell cycle_Regulation of G1/S transition (part 2)	9.534E-07	Cyclin A2, p107, Cyclin A, Cyclin E, CDK2, E2F1
**23**	Mitogenic action of Estradiol / ESR1 (nuclear) in breast cancer	9.534E-07	SGOL2, CDC25A, Cyclin E, Cyclin E2, CDK2, E2F1
**24**	DNA damage_ATM/ATR regulation of G1/S checkpoint	9.805E-07	PCNA, CDC25A, Chk2, Cyclin A, Cyclin E, Chk1, CDK2
**25**	DNA damage_Mismatch repair	2.202E-06	EXO1, WDHD1, PCNA, MutSalpha complex, MSH2, MSH6
**26**	Immune response_IFN-alpha/beta signaling via PI3K and NF-kB pathways	2.249E-06	PCNA, b-Myb, CDC25A, p107, Cyclin A, Cyclin E, CDK1 (p34), CDK2, E2F1

**27**	Higher ESR1 / ESR2 ratio in breast cancer	4.544E-06	Cyclin A2, CDC25A, Cyclin B1, SKP2, FOXM1, Cyclin E
**28**	HCV-dependent transcription regulation leading to HCC	4.608E-06	SKP2, HSP70, Cyclin E, CDK2, E2F1
**29**	IL-6 signaling in colorectal cancer	5.369E-06	Cyclin B1, HSP70, Cyclin B, Survivin, Cyclin E, CDK1 (p34)
**30**	Cell cycle_Role of 14-3-3 proteins in cell cycle regulation	5.904E-06	CDC25C, CDC25A, Chk2, CDK1 (p34), Chk1
**31**	Microsatellite instability in gastric cancer	5.904E-06	EXO1, PCNA, MutSalpha complex, MSH2, MSH6
**32**	Cell cycle_Regulation of G1/S transition (part 1)	6.311E-06	CDC25A, Chk2, Cyclin A, Cyclin E, Skp2/TrCP/FBXW, CDK2
**33**	Inhibition of remyelination in multiple sclerosis: regulation of cytoskeleton proteins	1.518E-05	chTOG, Tubulin beta, Tubulin alpha, Stathmin, Tubulin beta 4, Tubulin (in microtubules)
**34**	DNA damage_Brca1 as a transcription regulator	2.951E-05	PCNA, Cyclin B1, MSH2, Chk2, E2F1
**35**	Transcription_Ligand-dependent activation of the ESR1/SP pathway	2.951E-05	CDC25A, TYSY, Cyclin E, Cyclin E2, E2F1
**36**	dCTP/dUTP metabolism	3.381E-05	Small RR subunit, RRM1, Ribonucleotide reductase, RRM2, DNA polymerase theta, POLE2, [illegible]
**37**	Microsatellite instability in colorectal cancer	5.538E-05	EXO1, PCNA, MutSalpha complex, MSH2, MSH6
**38**	Rb proteins signaling in multiple myeloma	5.919E-05	p107, Cyclin E, CDK2, E2F1
**39**	Mechanisms of CAM-DR in multiple myeloma	6.397E-05	TOP2 alpha, HSP70, Cyclin A, Cyclin E, CDK2
**40**	Epigenetic alterations in ovarian cancer	6.580E-05	DNMT3B, Aurora-B, CDC20, SKP2, PARP-1, Aurora-A, EZH2

**41**	Brca1 and Brca2 in breast cancer	7.426E-05	BRIP1, MSH2, Rad51, Chk1
**42**	Cell cycle progression in Prostate Cancer	1.090E-04	CDC25A, Cyclin B, CDK1 (p34), CDK2, E2F1
**43**	Immune response_IL-4-induced regulators of cell growth, survival, differentiation and metabolism	1.102E-04	CDC25A, MCM6, MCM4, Cyclin A, Cyclin E, CDK2
**44**	Transcription_Role of heterochromatin protein 1 (HP1) family in transcriptional silencing	1.234E-04	Cyclin A2, CDC25A, Cyclin E, CDK1 (p34), E2F1
**45**	Proteolysis_Role of Parkin in the Ubiquitin-Proteasomal Pathway	1.940E-04	Tubulin beta, HSP70, Tubulin alpha, Cyclin E
**46**	DNA damage_Nucleotide excision repair	2.168E-04	Histone H2A, DNA polymerase kappa, PCNA, PARP-1, DTL (hCdt2), EZH2
**47**	The role of aberrations in CDKN2 locus and CDK4 in familial melanoma	3.599E-04	b-Myb, E2F2, p107, E2F1
**48**	Notch signaling in breast cancer	4.732E-04	Cyclin B1, HURP, Survivin, Cyclin A, CDK2
**49**	Cell cycle_Influence of Ras and Rho proteins on G1/S Transition	4.732E-04	Cyclin A2, SKP2, Cyclin E, CDK2, E2F1
**50**	IL-2 as a growth factor for T cells in multiple sclerosis	6.856E-04	PCNA, Cyclin E, CDK2, E2F1

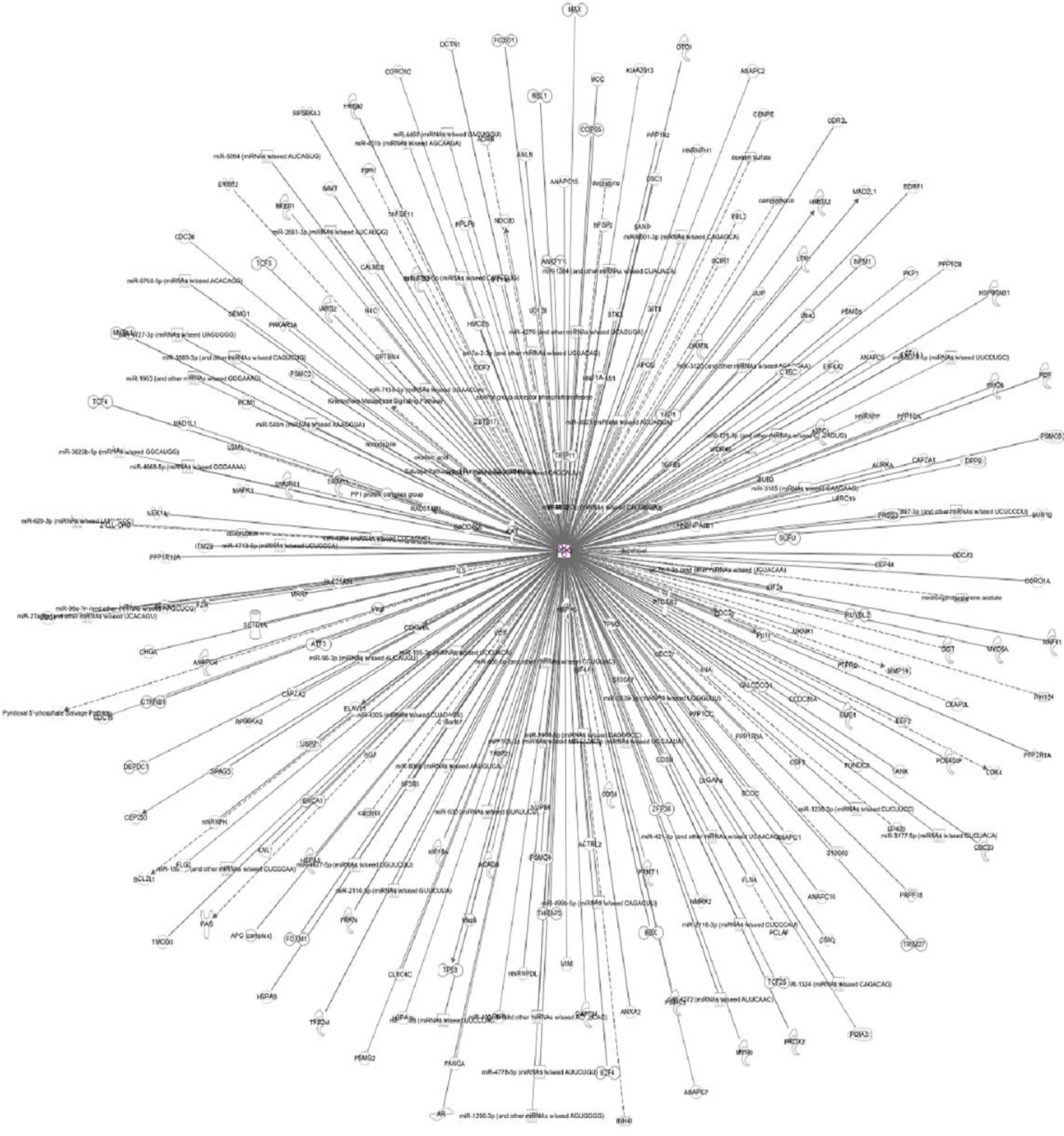

MAX
DCTN1
FOXO1
OTOF
KIAA2013
ANAPC2
CORO1C
RBL1
RPS6KA3
CENPE
CDR2L
HNRNPH1
ERBB2
IMMT
ANAPC15
RBL2
MAD2L1
EDRF1
CDC26
TCF3
NPM1
PPP1CB
PKP1
HSP90AB1
TCF4
MAD1L1
NEK1
PPP1R12A
STK3
JUP
TGFB1
AURKA
CAPZA1
CEP44
CORO1A
CHGA
CTNNB1
CAPZA2
RPS6KA2
DEPDC1
SPAG5
CEP250
HNRNPK
BRCA1
BCL2L1
FAS
APC complex
FOXM1
TMOD3
HSPA8
CLEC4C
TP53
HNRNPDL
VIM
PSMG2
FANCA
AR
INHA
ANXA2
ANAPC7
PDIA3
PRDX2
TCF25
TRIM27
ANAPC16
PRPF18
CDC23
CDK4
PPP2R1A
TANK
EEF2
NMRK2
PRMT1
FLNA
ZFP36
ACTBL2
PSMC4
SF3B1
USP7
ELAVL1
ATF3
SETD1A
MYO1A
RNF41
CDC20
SUFU
Pyridoxal 5'-phosphate Salvage Pathway
PP1 protein complex group
Kinetochore Metaphase Signaling Pathway
medroxyprogesterone acetate
camptothecin
dextran sulfate
decitabine
nocodazole
calcitriol

Figure S2. Analysis of micro (mi)RNA networks with NEK2 in breast cancer. We used the miRWalk database to identify associations with NEK2, and then network regulation was analyzed by Ingenuity Pathway Analysis (IPA). hsa-miR-1236-3p, hsa-miR-4264, hsa-miR-486-5p, hsa-miR-155-3p, and hsa-miR-6839-3p are co-expressed for breast cancer development.

Table S4. Correlation of NEK2 expression with the level of immune infiltration of B cells in BRCA (Analysis of TIMER database). rho shows the partial correlation value, and the level of statistical significance is shown with p-value and adjusted p-value. The threshold of significance was p-value <0.05.

Cancer	Infiltrates	rho	p-value	Adjusted p-value
**BRCA (n=1100)**	B cell memory_CIBERSORT	-0.072	0.023	0.084
**BRCA (n=1100)**	B cell memory_CIBERSORT-ABS	-0.062	0.051	0.148
**BRCA (n=1100)**	B cell memory_XCELL	0.045	0.158	0.333
**BRCA (n=1100)**	B cell naive_CIBERSORT	0.000	0.992	0.997
**BRCA (n=1100)**	B cell naive_CIBERSORT-ABS	0.049	0.126	0.288
**BRCA (n=1100)**	B cell naive_XCELL	0.109	0.001	0.005
**BRCA (n=1100)**	B cell plasma_CIBERSORT	-0.036	0.259	0.478
**BRCA (n=1100)**	B cell plasma_CIBERSORT-ABS	0.007	0.814	0.909
**BRCA (n=1100)**	B cell plasma_XCELL	0.052	0.100	0.246
**BRCA (n=1100)**	B cell_EPIC	0.019	0.550	0.750
**BRCA (n=1100)**	B cell_MCPCOUNTER	0.029	0.355	0.593
**BRCA (n=1100)**	B cell_QUANTISEQ	0.180	0.000	0.000
**BRCA (n=1100)**	B cell_TIMER	0.061	0.053	0.150
**BRCA (n=1100)**	B cell_XCELL	0.202	0.000	0.000
**BRCA (n=1100)**	Class-switched memory B cell_XCELL	0.101	0.001	0.011

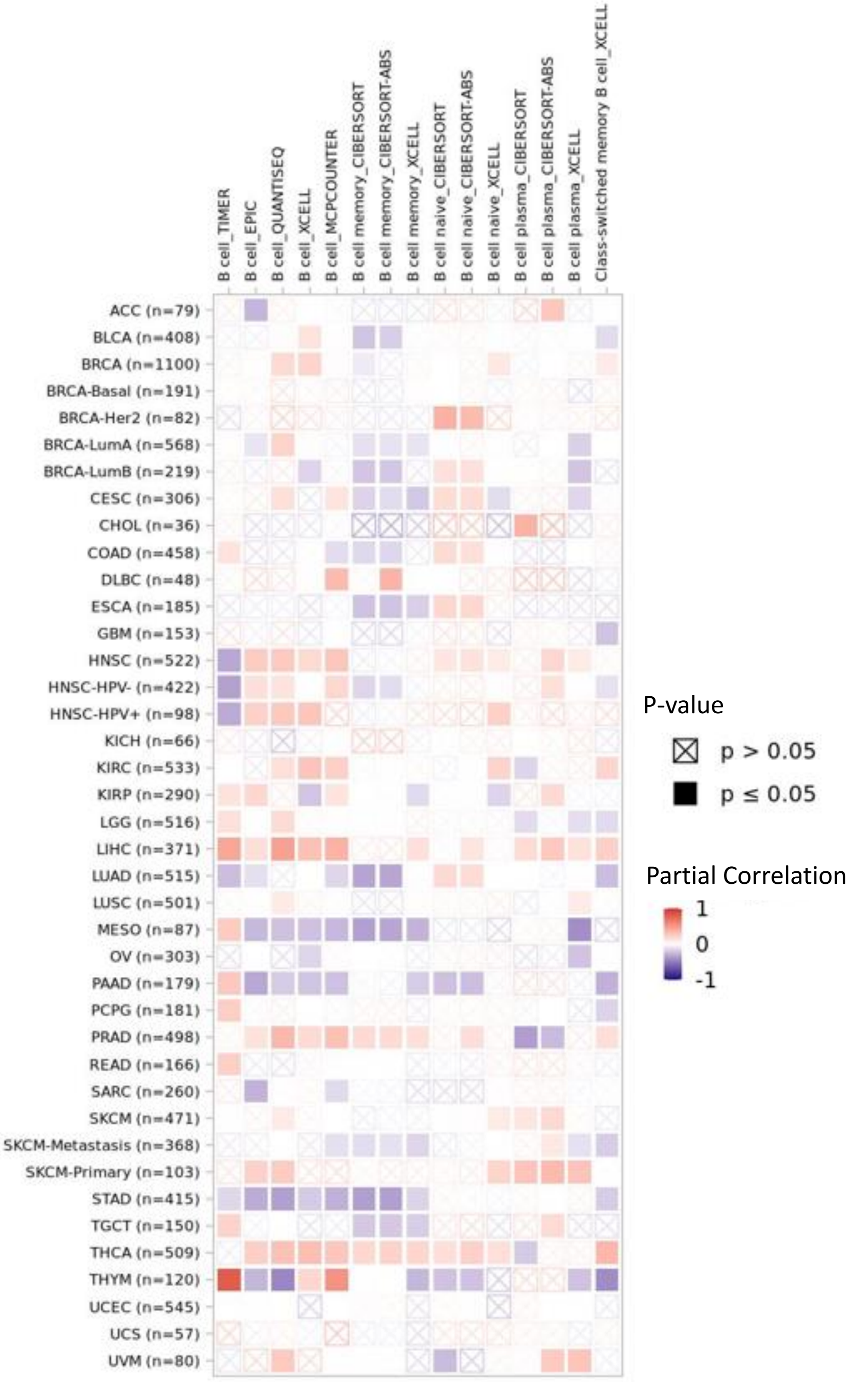

Figure S3. Heatmap of NEK2 expression with the level of immune infiltration of B cells in different types of cancer (Analysis of TIMER database). The partial correlation coefficient shows the size of the correlation, and the p-value indicates the level of statistical significance. The threshold of significance was p-value <0.05.

Table S5. Correlation of NEK2 expression with the level of immune infiltration of CD4+ T Cells in BRCA (Analysis of TIMER database). rho shows the partial correlation value, and the level of statistical significance is shown with p-value and adjusted p-value. The threshold of significance was p-value <0.05.

Cancer	Infiltrates	rho	p-value	Adjusted p-value
**BRCA (n=1100)**	T cell CD4+ (non-regulatory)_QUANTISEQ	-0.1357	0.0000	0.0001
**BRCA (n=1100)**	T cell CD4+ (non-regulatory)_XCELL	0.0401	0.2063	0.3350
**BRCA (n=1100)**	T cell CD4+ central memory_XCELL	-0.2947	0.0000	0.0000
**BRCA (n=1100)**	T cell CD4+ effector memory_XCELL	-0.2598	0.0000	0.0000
**BRCA (n=1100)**	T cell CD4+ memory activated_CIBERSORT	0.2223	0.0000	0.0000
**BRCA (n=1100)**	T cell CD4+ memory activated_CIBERSORT-ABS	0.2224	0.0000	0.0000
**BRCA (n=1100)**	T cell CD4+ memory resting_CIBERSORT	0.0617	0.0518	0.1188
**BRCA (n=1100)**	T cell CD4+ memory resting_CIBERSORT-ABS	0.1065	0.0008	0.0031
**BRCA (n=1100)**	T cell CD4+ memory_XCELL	0.2963	0.0000	0.0000
**BRCA (n=1100)**	T cell CD4+ naive_CIBERSORT	-0.0194	0.5406	0.6575
**BRCA (n=1100)**	T cell CD4+ naive_CIBERSORT-ABS	-0.0195	0.5399	0.6575
**BRCA (n=1100)**	T cell CD4+ naive_XCELL	-0.0297	0.3491	0.4943
**BRCA (n=1100)**	T cell CD4+ Th1_XCELL	0.1474	0.0000	0.0000
**BRCA (n=1100)**	T cell CD4+ Th2_XCELL	0.6766	0.0000	0.0000
**BRCA (n=1100)**	T cell CD4+_EPIC	0.0365	0.2496	0.3903
**BRCA (n=1100)**	T cell CD4+_TIMER	0.0045	0.8886	0.9314

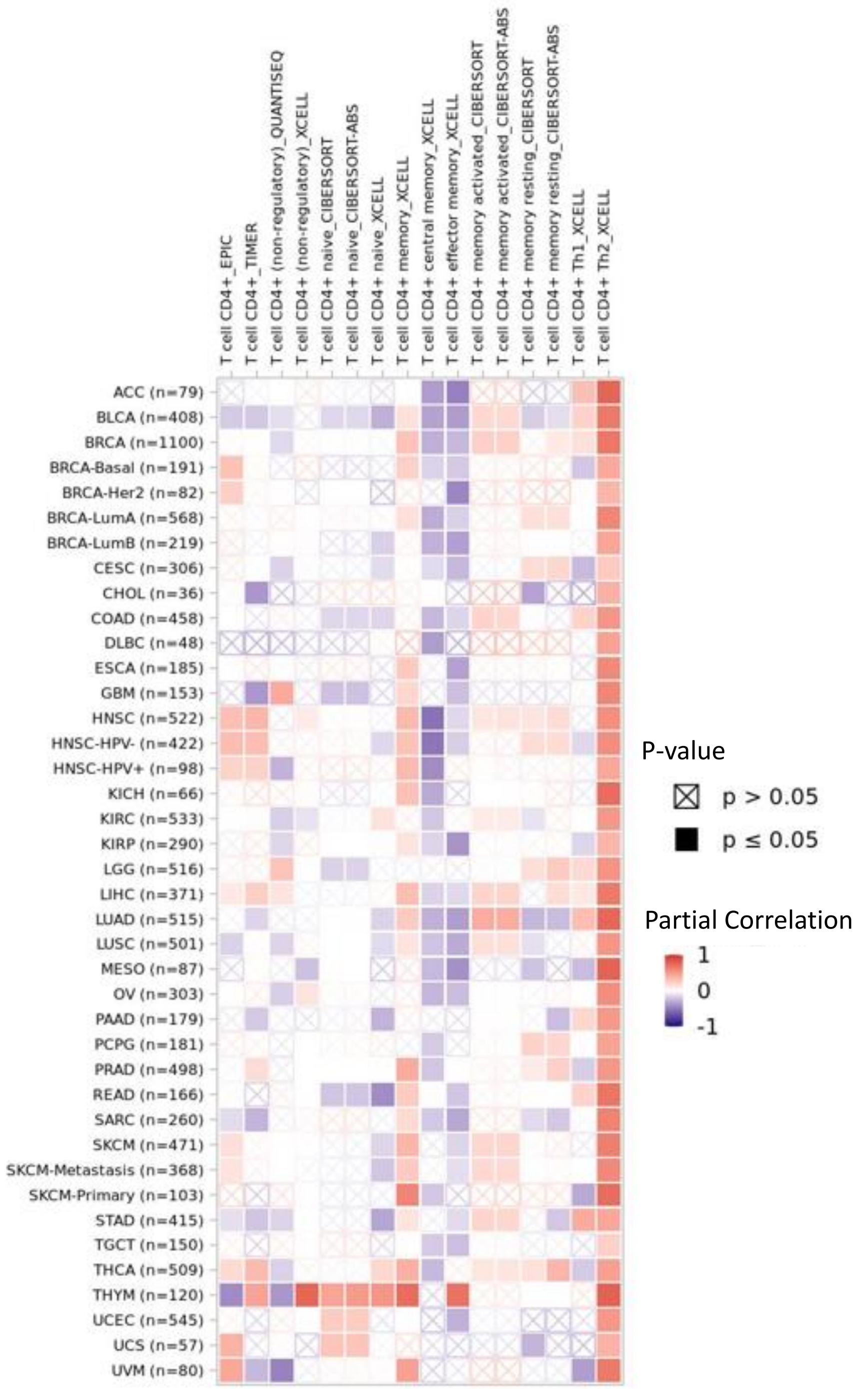

Figure S4. Heatmap of NEK2 expression with the level of immune infiltration of CD4+ T Cells in different types of cancer (Analysis of TIMER database). The partial correlation coefficient shows the size of the correlation, and the p-value indicates the level of statistical significance. The threshold of significance was p-value <0.05.
